# Dental and prosthodontic status of an over 40 year-old population in Shandong Province, China

**DOI:** 10.1186/1471-2458-11-420

**Published:** 2011-06-01

**Authors:** Qian Zhang, Dick J Witter, Ewald M Bronkhorst, Nico HJ Creugers

**Affiliations:** 1 Department of Prosthetic Dentistry, Affiliated Hospital of Medical School, Qingdao University, Jiangsu Road 16#, Qingdao, P.R. China; 2 Department of Oral Function and Prosthetic Dentistry, College of Dental Science, Radboud University Nijmegen Medical Centre, Philips van Leydenlaan 25, 6525 EX, Nijmegen, The Netherlands; 3 Department of Preventive and Restorative Dentistry, College of Dental Science, Radboud University Nijmegen Medical Centre, Philips van Leydenlaan 25, 6525 EX, Nijmegen, The Netherlands

## Abstract

**Background:**

This study aims to (1) describe the dental status using DMFT for the whole dentition and the anterior, premolar and molar regions; (2) determine associations of demographic variables and socio-economic status (SES) with DMFT and tooth replacement; (3) analyze to what extent the goal as proposed by the WHO -'the retention of not less than 20 teeth throughout life' is achieved.

**Methods:**

DMFT and tooth replacement data of 1588 subjects over 40 years from urban and rural sites in Qingdao (Shandong Province, China) were collected. Relative D, M, and F scores per dental region were calculated and compared by paired T-tests. Multivariable logistic regression was used to determine relationships with age, gender, place of residence, and SES.

**Results:**

Mean numbers of D and F were low (1.36 respectively 0.27) at all ages. Molars had highest chance for D and M. For the molar region every additional year of age gave significantly lower chance for D and higher chance for M (OR: 0.98 and 1.02 respectively; both p ≤ 0.01). Mean number of M was associated with age (approximately 1.5 in each jaw at 40 years and 6 at 80 years). Females had higher chance for D (OR: 1.34; p ≤ 0.05) and F (OR: 1.69; p ≤ 0.01), and lower chance for M (OR: 0.60; p ≤ 0.01). Urban and rural subjects had similar chance for D; urban subjects had approximately 5 times more chance for F (p ≤ 0.01). SES had no relationship with D and M, however SES low was associated with F (OR: 0.45; p ≤ 0.01). Replacements were significantly associated with age (all dental regions except anterior region), gender (all dental regions), place of residence (whole dentition and molar region), and SES (whole dentition and premolar and molar regions).

**Conclusions:**

The majority of subjects presented a reduced dentition. Molars were most frequently affected by D and M. D, M, F and replaced teeth were associated with the background variables, however differently for different dental regions. Above the age of 70 years, only 64% of the subjects presented 'not less than 20 natural teeth'.

## Background

In Mainland China only few studies reported on the dental health status of the adult population, the largest of them conducted in Beijing, Guangdong, and Hong Kong before the year 2000 [1-3]. More recent dental health surveys in China focused on the prevalence of root caries and presented little or no information about other dental health outcomes such as tooth loss [4-6]. Tooth loss is an important predictor for oral health-related quality of life[7].

The mostly used index to register dental health status in epidemiological studies is the decayed/missing/filled teeth (DMFT) recording system. The index is used as an indicator to describe dental diseases in terms of decayed and missing teeth and an estimate of dental care by means of filled teeth, but provides no information with regards to the (remaining) functionality of dentitions. The complement of the 'missing' component in DMFT - number of present teeth - is often used to assess the functionality of dentitions. Unfortunately, DMFT does not provide information regarding the type of missing teeth or whether they are functional in occlusion. This is of special interest with adults over 40 years who show an increasing number of missing teeth with aging as was demonstrated in a recent systematic review [8]. It was recommended for future reports to include not only the number of missing teeth but also additional information regarding the location of missing teeth and, if possible, information regarding tooth replacements to describe functionality. A recently published systematic review provided evidence that location and distribution of tooth loss is associated with impairment of oral health-related quality of life as well as with the severity of the impairment [7]. Another systematic review emphasized the need to describe the status of teeth in the different dental regions for assessing oral function [9]. To date no information is available regarding tooth loss in different dental regions in Chinese adults.

It was the purpose of this study to describe the dental and prosthodontic status of a Chinese population over 40 years of age using DMFT not only at dentition level but also for the three dental regions (anterior, premolar and molar regions). Moreover, we aimed to determine possible associations between various demographic and socio-economic factors on dental status and tooth replacements, and to determine the chance of teeth for being decayed, missing, or filled. Finally, it was the purpose of this study to find out to what extent the goal as proposed by the WHO - the retention of not less than 20 teeth throughout life -, without or with prosthetic replacement, is met in China.

## Methods

The study was conducted in the Qingdao area, located at the east coast of Shandong Province (population 94 million in 2008), Eastern China. Qingdao City (population 3 million) has direct jurisdiction over the surrounding rural territory, including 5 county-level cities. Each rural county comprises 40 - 80 small rural villages. Qingdao area (urban and rural) has approximately 8 million inhabitants.

### Sampling method

For this study a cross-sectional survey, representing 1588 subjects aged ≥ 40 years living in urban and rural areas in Qingdao, Shandong Province, was conducted (Table 1). To calculate the sample size needed, it was decided that the sample should allow for multiple logistic regression with at least 12 independent variables amongst dentate subjects. This implies that at least 120 observations of the least prevalent part of a dichotomous variable amongst dentate subjects are necessary. Using 8% prevalence as a worst-case scenario, a total sample size of 1500 is needed to attain the 120 observations needed. To allow for an estimated 5% prevalence of edentulous subjects, the targeted population was increased to 1575.

**Table 1:** Number (%) of included subjects (n = 1588) according to gender and place of residence

	Urban	Rural	Total
Female	418 (51.2)	381 (49.4)	799 (50.3)
Male	398 (48.8)	391 (50.6)	789 (49.7)
Total	816 (51.4)	772 (48.6)	1588 (100)

Subjects were selected randomly from administrative lists of residents of communities or villages provided by local authorities and lists of employees of factories. Inclusion aimed at proportional distribution according to age, gender and place of residence (urban or rural). Data were collected in 2009 and 2010.

The urban sample was constructed after consulting local authorities on the basis of accessibility, and comprised 11 communities and 4 factories in Qingdao City. Administrators of the communities informed and invited their residents for participation in this study. The examination venue usually was a neighbourhood community office or a social centre for elderly. A total of 570 community inhabitants and 193 employees from factories were included on the basis of voluntary participation. As truly representative sampling was not feasible the pathfinder sampling method was adopted incorporating sufficient examination sites to cover relevant groups of the population intended [10]. It appeared that subjects of certain age groups were underrepresented in the initial urban sample (mostly males). Therefore, a complementary convenient sub-sample, drawn from community residents attending a health centre while they were waiting for a periodical check-up, was eventually included. Fifty-three subjects were included in this way.

For the rural sample, one county (Zhugou) considered representative for northeast Shandong Province was chosen on the basis of accessibility for investigating dental health status and cooperation from local authorities. This county (a predominantly agrarian area with a low population density and a total population of approximately 36,000) is located approximately 120 km northwest from Qingdao City and comprises 56 villages ranging from 153 to 1583 inhabitants. On the basis of information from the local authorities it appeared that there were large differences in income amongst the villages. As Gross Domestic Product (GDP) was expected to be related with socio-economic status (SES), 10 villages with different GDP were selected randomly: 3 villages out of 19 with highest 2008 GDP; 4 out of 18 with middle GDP, and 3 out of 19 with lowest GDP. Next subjects from these villages were randomly selected using administrative name-lists. In cases where subjects were invited but did not show up (n = 347; 45%), other subjects were randomly drawn from the same sampling lists.

The research was carried out in compliance with the Helsinki Declaration and was approved by the ethics committee of the medical school at Qingdao University, Qingdao, China.

### Interview and clinical examination

The fieldwork included an interview and a clinical oral examination. After obtaining verbal consent from the participants, a structured questionnaire was completed. The questionnaire was used previously in a study in Vietnam [11] and was translated into Mandarin. This Chinese version was checked for language adequacy by a panel of dentists and pilot tested on 20 Chinese subjects to assess clarity. Some minor modifications were made based on the results of the pilot. Subjects that needed assistance in completing the questionnaire, i.e. because of (functional) illiteracy or visual impairment, were helped by a dental assistance. If needed they read aloud the questions and recorded the answers. After completion, the questionnaire was checked for unrecorded items, and if applicable, subjects were requested to complete the form. For the present study only answers regarding SES were used.

For assessment of SES (high, middle, low) a modified Kuppuswamy classification was used [12], which is based on the subject's level of education (5 levels: higher education; college; primary school; no formal education, literate; no formal education, illiterate), occupation (3 levels: white collar = office worker, teacher, doctor and academic researcher, government officers; service people = salespeople, house worker and vehicle driver; blue collar = farmer, factory worker, forestry worker, fisher and (lower) military personal), and household income (4 levels: income covers expenses, no loans needed; income does not cover expenses, no loans needed; income covers expenses, loans needed incidentally; income does not cover expenses' loans needed regularly).

Next, subjects received an oral examination. A calibrated dentist who was calibrated against experienced researchers conducted the examination under natural light. Inter-observer agreements for assessing decayed, missing, and filled teeth were excellent (kappa's ≥ 0.89). An overhead light was used when there was insufficient natural light. Procedures and diagnostic criteria recommended by the World Health Organization were applied [13]. Teeth were neither cleaned nor dried before clinical examination, but food debris obscuring visual inspection was removed. Caries was assessed by visual inspection and with additional tactile inspection by a probe if required. Where any doubt existed, no caries was recorded. 'Filled' teeth with secondary caries were recorded as 'decayed' as well. Present teeth without being decayed or filled were considered 'sound' (S). Roots were considered in the analyses in two different ways. According to the WHO criteria, for DMFT calculations a root was considered as a decayed tooth. In the analysis of tooth replacements, roots were considered non-functional candidates for replacement and therefore considered as missing teeth. Tooth replacements (R) were recorded as such when teeth were replaced either by fixed dental prostheses or removable dental prostheses.

### Data analysis

Of all recorded variables only tooth status 'decayed' (D), 'missing' (M), 'filled' (F), sound (S), and replacements of missing teeth (R) were used in the present analyses. Edentulous subjects were excluded from this analysis. The mean numbers of D, M, F, and S of the subjects were plotted against age for the whole dentition as well as for the anterior, the premolar and the molar regions separately.

Initially, multivariable logistic regression analyses were performed separately for upper and lower jaw to determine relationships between the background variables age, gender, place of residence, and SES with the distribution of non-sound teeth over D, M and F, i.e. D_ratio _= D/(D+M+F), M_ratio _= M/(D+M+F), F_ratio _= F/(D+M+F). Since these distributions were skewed, the ratios were dichotomized using the following cut-off points: D_ratio_: 0 = no decayed teeth; 1 = one or more decayed teeth present; M_ratio_: 0 = no missing teeth; 1 = one or more teeth missing; F_ratio_: 0 = no filled teeth present; 1 = one or more filled teeth present. It appeared that associations, if present, were always in the same direction. Therefore, the regression analyses of upper and lower jaw were combined. In all multiple regression models only theoretical considerations were used to select the independent variables in the models. So statistical methods to select "strongest" variables, such as backward of forward selection were not applied.

Relative D, M, F and S scores per dental region (D_rel_, M_rel_, F_rel_, and S_rel_) were determined by dividing the number of teeth having the respective status (i.e. decayed) by the total number of teeth concerned in each region. Mean D_rel_, M_rel_, F_rel _and S_rel _of each dental region were compared by paired T-tests.

2Possible associations of background variables were also analyzed for tooth replacements (R). Replacement ratio was defined as R_ratio _= R/(M + root(s)) and dichotomized with cut-offs: 0 = no tooth replaced; 1 = one or more missing teeth replaced. The percentage of dentate subject having tooth replacements was plotted for urban and rural residence according to age groups (40-49 yrs; 50-59 yrs, 60-69 yrs, and ≥ 70 yrs).

## Results

Of the total sample (n = 1588), 63 (4%) subjects were edentulous. The remaining 1525 subjects were included in the statistical analysis.

### Decayed teeth

**Figure 1:**
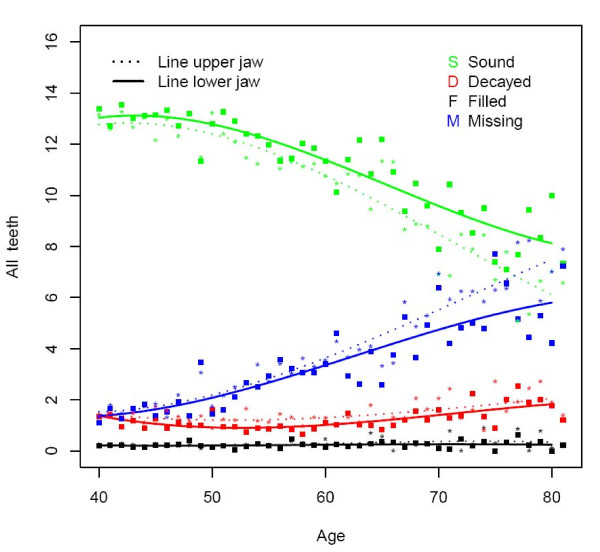
**Mean number of decayed (D), missing (M), filled (F), and sound (S) teeth by age (n = 1525)**.

**Figure 2:**
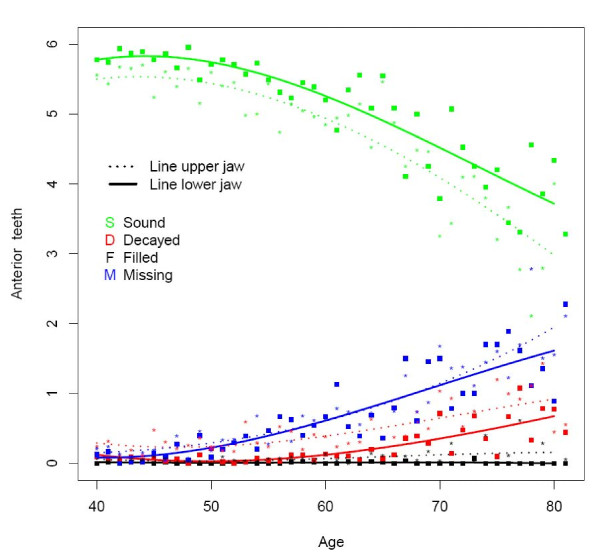
**Mean number of decayed (D), missing (M), filled (F), and sound (S) teeth in the upper and lower anterior region by age (n = 1525)**.

**Figure 3:**
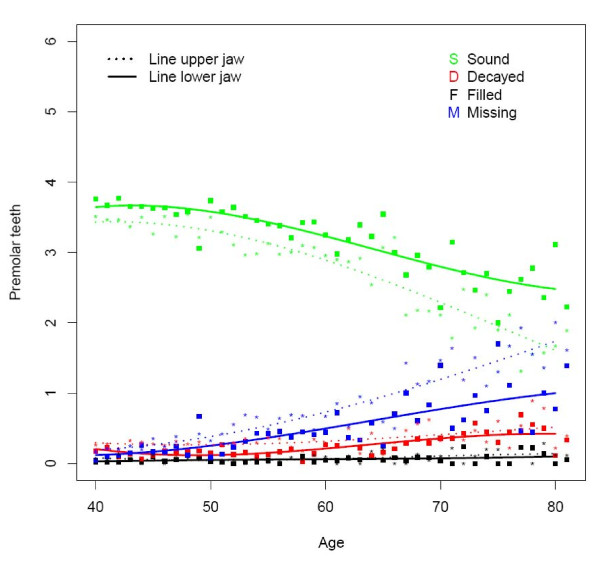
**Mean number of decayed (D), missing (M), filled (F), and sound (S) teeth in the upper and lower premolar region by age (n = 1525)**.

**Figure 4:**
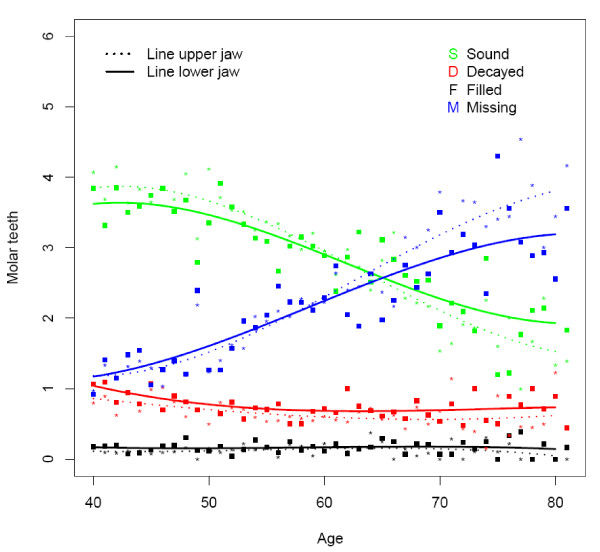
**Mean number of decayed (D), missing (M), filled (F), and sound (S) teeth in the upper and lower molar region by age (n = 1525)**.

The majority of subjects presented one or more decayed teeth, being slightly higher in rural (78%) than in urban areas (74%) (Table 2). At all ages the overall D component was more or less similar for the upper and lower jaw (Figure 1). This can be seen in all dental regions except for the anterior region where the D component was higher in the upper jaw (Figures 2, 3 and 4). The mean number of decayed teeth was highest for the molar region compared to the anterior and premolar regions, but this difference diminished for subjects aged over 60.

**Table 2:** Percentage of dentate subjects (n = 1525) with decayed, missing and filled teeth

	Number of subjects by age category	Percentage of subjects		
		
		Decayed teeth	Missing teeth	Filled teeth
Rural				
40-49	299	77.3	84.3	10.0
50-59	189	78.3	94.7	6.3
60-69	135	76.3	97.0	7.4
≥70	93	81.7	100	4.3
Urban				
40-49	247	72.9	81.4	36.4
50-59	253	73.1	90.9	34.8
60-69	209	75.1	91.9	42.6
≥70	100	78.0	97.0	43.0

Logistic regression analysis (Table 3) shows that each additional year of age gives a lower chance for having decayed molars (OR = 0.98; p = 0.002; over a 5 year period: OR = 0.92). Significant associations between age and decay could not be demonstrated for the other dental regions. Females had a higher chance for having decayed teeth (OR = 1.34; p = 0.02). The chance for having decayed teeth was not associated with place of residence or SES.

**Table 3:** Odds ratios of D_ratio_, M_ratio_, F_ratio _and R_ratio_, 95% confidence intervals (CI) for adjusted odds ratios, and level of significance for the whole dentition, and for the anterior, premolar and molar regions separately (n = 1525)

	D			M			F			R		
	
	Unadjusted OR	Adjusted OR	95% CI	Unadjusted OR	Adjusted OR	95% CI	Unadjusted OR	Adjusted OR	95% CI	Unadjusted OR	Adjusted OR	95% CI
**All regions**												
Age	1.00	1.00	[0.99 - 1.01]	0.99	0.99	[0.98 - 1.01]	1.01	1.01	[0.99 - 1.02]	***1.04***	***1.05***	[1.04 - 1.06]
Female^a^	**1.33**	**1.34**	[1.04 - 1.72]	***0.60***	***0.60***	[0.45 - 0.79]	***1.67***	***1.69***	[1.30 - 2.19]	***1.48***	***1.67***	[1.33 - 2.09]
Urban^b^	0.78	0.77	[0.56 - 1.05]	0.79	0.90	[0.63 - 1.28]	***7.48***	***5.34***	[3.68 - 7.74]	***0.71***	***0.57***	[0.42 - 0.76]
SES-high^c^	0.89	1.13	[0.83 - 1.55]	0.84	0.88	[0.61 - 1.27]	2.74	0.92	[0.68 - 1.25]	0.73	0.86	[0.64 - 1.15]
SES-low^c^	1.33	1.18	[0.84 - 1.66]	1.06	1.03	[0.71 - 1.49]	***0.21***	***0.45***	[0.29 - 0.69]	**1.28**	**0.70**	[0.51 - 0.95]
**Anterior region**												
Age	1.00	1.00	[0.98 - 1.01]	1.00	1.00	[0.99 - 1.02]	1.00	1.00	[0.97 - 1.02]	1.00	1.00	[1.00 - 1.02]
Female^a^	***1.89***	***1.82***	[1.32 - 2.50]	***0.47***	***0.49***	[0.35 - 0.68]	1.59	1.60	[0.96 - 2.67]	**1.44**	**1.52**	[1.02 - 2.28]
Urban^b^	0.79	0.98	[0.64 - 1.50]	0.75	0.69	[0.45 - 1.07]	***9.97***	***5.16***	[2.24 - 11.91]	1.23	1.04	[0.62 - 1.75]
SES-high^c^	0.66	0.76	[0.50 - 1.16]	0.94	1.10	[0.71 - 1.71]	4.04	1.25	[0.71 - 2.21]	1.17	1.03	[0.59 - 1.78]
SES-low^c^	1.57	1.21	[0.79 - 1.85]	0.94	0.91	[0.59 - 1.40]	**0.12**	**0.35**	[0.13 - 0.98]	0.83	0.77	[0.46 - 1.28]
**Premolar region**												
Age	0.99	0.99	[0.98 - 1.01]	1.01	1.01	[1.00 - 1.02]	0.99	1.00	[0.97 - 1.01]	***1.03***	***1.03***	[1.02 - 1.05]
Female^a^	1.08	1.05	[0.80 - 1.38]	0.82	0.87	[0.65 - 1.16]	1.54	1.47	[0.97 - 2.23]	***2.06***	***2.35***	[1.70 - 3.25]
Urban^b^	**0.63**	**0.70**	[0.49 - 1.00]	0.72	0.72	[0.49 - 1.04]	***22.16***	***16.58***	[7.33 - 37.50]	1.04	0.85	[0.56 - 1.29]
SES-high^c^	0.77	1.15	[0.80 - 1.65]	0.73	0.78	[0.53 - 1.14]	3.97	1.01	[0.63 - 1.62]	0.85	0.77	[0.49 - 1.18]
SES-low^c^	1.54	1.38	[0.95 - 2.01]	1.07	0.76	[0.53 - 1.12]	0.13	0.54	[0.24 - 1.20]	**0.99**	**0.58**	[0.38 - 0.88]
**Molar region**												
Age	***0.90***	***0.94***	[0.97 - 0.99]	***1.02***	***1.02***	[1.01 - 1.03]	1.00	1.00	[0.99 - 1.01]	***1.04***	***1.05***	[1.04 - 1.06]
Female^a^	1.15	1.15	[0.92 - 1.44]	***0.70***	***0.69***	[0.54 - 0.68]	***1.73***	***1.69***	[1.27 - 2.23]	***1.52***	***1.71***	[1.34 - 2.18]
Urban^b^	0.92	0.90	[0.68 - 1.20]	0.70	0.80	[0.59 - 1.09]	***6.55***	***5.11***	[3.38 - 7.74]	***0.68***	***0.55***	[0.40 - 0.75]
SES-high^c^	1.0	1.1	[0.82 - 1.46]	0.74	0.90	[0.66 - 1.24]	2.42	0.89	[0.64 - 1.23]	0.71	0.86	[0.63 - 1.18]
SES-low^c^	1.0	1.05	[0.77 - 1.42]	1.42	1.18	[0.86 - 1.63]	***0.25***	***0.52***	[0.32 - 0.84]	**1.37**	**0.72**	[0.52 - 1.00]

Bold = *P *≤ 0.05; Bold & Italic = *P *≤ 0.01 Reference (OR = 1) respectively: a male, b rural, c SES middle

### Missing teeth

The percentage of subjects having at least one missing tooth was lowest for the youngest urban age group (81.4), while in the oldest rural age group all subjects presented at least one missing tooth (Table 2). The mean number of missing teeth varied from 1.3 in each jaw at the age of 40 to 5.7 in each jaw at the age of 80 (Figure 1). From the age of 60, the mean number of missing teeth in the upper jaw was higher than in the lower, which is best demonstrated in the premolar and molar region (Figures 2, 3, and 4).

Logistic regression analysis confirmed the significant association of missing teeth in the molar region with age (OR = 1.02, p = 0.001) (Table 3). Every additional year of age resulted in a 1.8% higher chance for having missing teeth in this region. Females had significantly lower chance for having missing anterior (OR = 0.49; p < 0.0001) and molar teeth than males (OR = 0.69; p = 0.003). Place of residence and SES was not associated with M_ratio_.

### Filled teeth

In the rural area the percentage of subject having at least one filled tooth was less than 10 whereas this percentage was over 34.8 in the urban area (Table 2). The mean number of filled teeth was low (≤0.27) for all ages and in all dental regions (Figures 1, 2, 3, and 4). Urban citizen had a 5.34 times higher chance for having fillings than rural residents (Table 3). This higher chance was most prominent for premolar teeth (OR = 16.58; p < 0.0001). A gender effect was seen for the whole dentition: females had a significantly higher chance for having filled teeth than males, especially in the molar region (OR = 1.69; p < 0.0001). Subjects in the category SES low had significantly fewer filled teeth (OR: 0.45; p < 0.001), except for the premolar region.

### Sound teeth

The mean number of sound teeth (S) in each jaw varied from 13.2 for 40 years old subjects to 6.6 in the upper and 8.3 in the lower jaw for subject at the age of 80 (Figure 1). The anterior and premolar regions showed a higher mean number of sound teeth in the lower jaw compared to the upper (Figures 2 and 3).

### Relative scores for decayed, missing and filled teeth per dental region

Molars showed significant higher chance for being decayed and missing when compared to premolar and anterior teeth (Table 4). Differences in the chance for having filled teeth were, although significant in 3 out of 4 comparisons, relatively small amongst the dental regions.

**Table 4:** Relative scores (%) for decayed, missing, and filled teeth in molar (M), premolar (PM), and anterior (A) dental region and mean difference (%) of relative scores between the dental regions (n = 1525)

Relative scores	%	Comparison	Mean difference (%)	95% CI	P value
*Upper jaw *					
Decayed					
M	11.2				
PM	7.8	M-PM	3.4	2.3 ... 4.4	< 0.001
A	6.2	M-A	5.0	4.0 ... 6.0	< 0.001
Missing					
M	33.8				
PM	15.7	M-PM	18.1	16.9 ... 19.3	< 0.001
A	9.3	M-A	24.5	23.2 ... 25.8	< 0.001
Filled					
M	2.1				
PM	1.7	M-PM	0.4	-0.1 ... 0.9	0.164
A	1.1	M-A	1.0	0.6 ... 1.5	< 0.001
*Lower jaw*					
Decayed					
M	12.9				
PM	5.2	M-PM	7.7	6.7 ... 8.6	< 0.001
A	2.5	M-A	10.4	9.4 ... 11.4	< 0.001
Missing					
M	32.6				
PM	10.2	M-PM	22.4	21.2 ... 23.6	< 0.001
A	8.5	M-A	24.1	22.7 ... 25.5	< 0.001
Filled					
M	2.7				
PM	1.3	M-PM	1.4	0.9 ... 1.9	< 0.001
A	0.1	M-A	2.6	2.1 ... 3.0	< 0.001

### Teeth replaced

**Figure 5:**
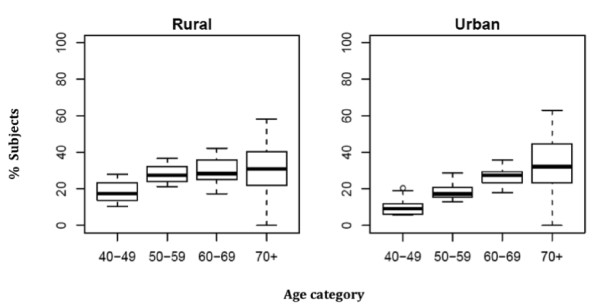
**Percentage of subjects dentate in both jaws (n = 1462) having replaced teeth by age category and place of residence**.

R_ratio _was associated with all background variables, however, differently for different dental regions: age was significant for all dental regions except for the anterior region, gender was significant for all dental regions, place of residence for the whole dentition and for the molar region, and SES for the whole dentition and the premolar and molar regions (Table 3). In general, the chance for having teeth replaced was higher for every additional year of age (OR = 1.05; p < 0.05), females had higher chance to have their teeth replaced (especially in the premolar region; OR = 2.35; p < 0.001), and urban residents had their teeth less often replaced than rural residents (OR = 0.57; p < 0.01). The distribution of missing teeth replaced (Figure 5) also shows that fewer replacements were found in urban than in rural residents. Subjects in the category SES low had a lower chance for having teeth replaced. In urban areas the mean number of teeth eligible for replacement (missing teeth and roots) ranged from 3.9 to 10.1 (Table 5). In rural areas these figures ranged from 5.1 to 18.7. However, in rural residents the percentages of actually replaced teeth were higher in the two youngest age categories (18.2 respectively 28.8 compared to 10.9 respectively 19.0 in urban residents). It appeared that these tooth replacements were predominantly removable dental prostheses. In urban subjects 79% of anterior replacements were removable dental prostheses compared to 68% in rural subjects. For premolar and molar replacements these percentages were respectively 73 and 66 in urban subjects and 62 and 53 in rural subjects.

**Table 5:** Number (%) of subjects dentate in each jaw (n = 1462) with missing teeth, mean number of missing teeth (SD) eligible for replacement, and mean percentage (SD) of teeth replaced according to age groups.

	Number of subjects dentate in each jaw with missing teeth (%)	Mean number of missing teeth (SD) eligible for replacement	Mean percentage (SD) of teeth replaced
Rural			
40-49	267 (89)	5.1 (4.0)	18.2 (28.4)
50-59	183 (97)	8.4 (6.7)	28.8 (33.5)
60-69	133 (99)	12.3 (9.0)	29.5 (32.3)
≥70	93(100)	18.7 (8.4)	33.1 (34.4)
Urban			
40-49	214 (87)	3.9 (3.1)	10.9 (25.5)
50-59	232 (92)	5.1 (3.9)	19.0 (28.6)
60-69	192 (92)	7.0 (5.7)	26.5 (32.4)
≥70	98 (98)	10.1 (7.2)	33.3 (33.9)

**Figure 6:**
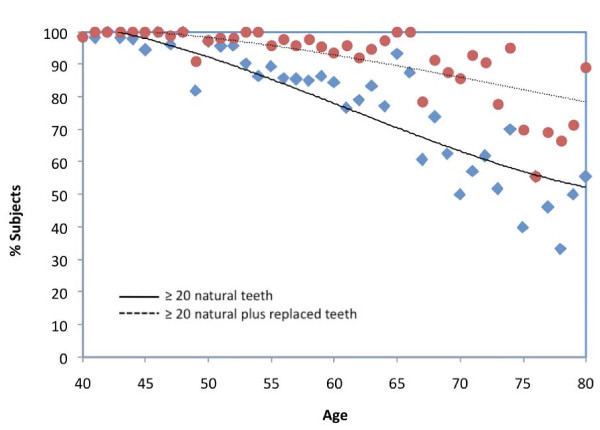
**Percentage of subjects dentate in each jaw (n = 1462) with at least 20 natural teeth or with at least 20 natural plus replaced teeth**.

The percentage of subjects dentate in both jaws that presented at least 20 natural teeth varied from 100% at the age of 43 years to 64% at the age of 70 (Figure 6). When replaced teeth are taking into account (at least 20 natural plus replaced teeth), these percentages varied from 100 at the age of 47 years to 88 at the age of 70.

## Discussion

This study aimed to investigate the dental and prosthodontic status of adults living in rural and urban areas in Qingdao, Shandong province, China. The inclusion of subjects aimed at proportional distribution according to place of residence, gender and age. With the aid of the local governmental administrative system this goal was reasonably well met in the rural area; therefore the rural sample is considered to reflect the rural population of Shandong Province. In the urban area inclusion of the intended subjects through administrative lists appeared to be more complicated. To deal with this, a pathfinder sampling method was used to find subjects from randomly chosen communities and factories. Eventually unfilled cells were filled with community residents attending a health centre for periodical check-up. Although the composition of this convenient sub-sample (which is 6% of the total urban sample) appeared to be slightly different from the total urban sample with respect to SES and gender (i.e. males above the age of 70 were not represented in the sub-sample), we consider that the urban sample reflects the population of Qingdao City.

Analyses of relationships between dental and prosthodontic status on the one hand and demographic and SES variables on the other hand are scarce for China [14].

SES is a complex construct often estimated by a combination of several indicators. Kuppuswamy's SES classification, whether or not modified, has been recommended for community-based research [12]. As this classification includes both the individual as well as the family socio-economic status, it is considered suitable for assessing SES in China.

In this study, the percentage of edentulous subjects was 4%, which is comparable with previous reports [1,2].

Until the late 1980's it was considered that in Mainland China decayed teeth were more prevalent in urban than in rural areas [15]. The first study reporting higher prevalence of decayed teeth in rural areas was published in 1989 (Luan et al.)[1], stating that, depending on age, the prevalence of one or more decayed or filled teeth ranged from 48 to 90% in urban residents, and from 51 to 97% in rural residents. This picture was confirmed by a study from 2001 in which people living in rural areas had a higher D-score than those living in urban areas (2.2 vs. 1.2 for subjects aged 35-44 years and 4.2 vs. 2.7 for subjects aged 65-74)[16]. The present study did not demonstrate significant differences in decayed teeth for place of residence.

However, the percentage of subjects presenting decayed teeth (ranging from 72.9% for urban residents aged 40 to 49 to 81.7% for rural residents aged 70 or more) was higher than in recent studies from other parts of China (64-67% [4,5]) but lower compared to other countries in Asia (82-92% in Delhi, India [17]), 90% in Sri Lanka [18], and 84-91% in Thailand [19]).

In this study SES had no relationship with decayed and missing teeth, but subjects with SES low had less chance to have filled teeth and also less chance for having teeth replaced, except for anterior teeth. However, compared to the variable 'place of residence', SES appeared to be a much less influential for fillings, indicating that accessibility to dental care might be of more importance as affordability.

The high prevalence of decay in rural subjects might be related to the economic development of the rural area over the last two decennia. Residents in both rural and urban areas today have similar access to cariogenic food, but in rural areas preventive programs are lacking and dental health knowledge is low [5,20,21]. However, urban residents showed an almost 5 times higher chance for having their teeth filled than rural residents. The combination of a high prevalence of decay on the one hand and a low prevalence of filled teeth and a high prevalence of missing teeth on the other hand indicates that tooth extraction is still the main treatment for dental diseases in rural areas. This might be because of the restricted accessibility to preventive dental care and lack of well-trained dental personnel in the rural region. An additional reason for the low number of fillings in rural areas might be higher cost of restorative care compared to tooth extraction.

The differences found in this study amongst dental regions underline the importance to differentiate between dental regions [9]. In a review it has been stated that aesthetics and patient satisfaction are markedly impaired with loss of anterior teeth, whilst satisfaction is most likely to be achieved in subjects with a premolar dentition. In this study the molar region was significantly more affected by decay and tooth loss than premolar and anterior regions. However, the premolar region in the upper jaw showed more often missing teeth than in the lower jaw.

As previously reported in a review of oral health surveys in China, gender appeared to be associated with DMFT [14]. In the sample of this study, females had higher chance for having decayed teeth, but lower chance for missing. On the other hand they had higher chance for filled teeth and tooth replacements than males. This higher grade of utilization of dental care amongst woman has been reported earlier [20].

The proportion of subjects with missing teeth replaced was higher than was assumed on the basis of a systematic review on dental health status and prosthodontic conditions of Chinese adults [8]. This high proportion of replacements was not only seen in urban residents but also in rural residents. Removable dental prostheses were more often found among urban subjects than among rural subjects, whereas the reverse tendency was seen for fixed dental prostheses. The same finding has been reported in earlier studies conducted in Beijing [22] and Guangdong Province [23]. The explanation presented by the authors of these studies was that in rural areas, many dental care providers have been trained in traditional apprenticeships rather than at university level dental schools, and mainly provide pain relief by tooth extraction followed by prosthetic treatment [23]. For common dental problems caused by caries or periodontal diseases, these providers prefer to extract involved teeth, above treatments that would involve the retention of such teeth [23]. Moreover, they seem to practice often rather unconventional prosthodontic principles, in which they tend to provide fixed dental prostheses for low prices rather than removable dental prostheses, even when only very few teeth are available as abutment teeth. The present data suggest that this explanation is also valid for the rural areas of Shandong Province today.

The percentage of subjects dentate in both jaws that presented at least 20 natural teeth (100% at 40 years and 64% at 70) was higher than in Vietnamese adults (88% at 40 years and 35 at 70 [11]) but similar to a Swedish cohort of subjects aged 70 (65%) [24]. As in these studies and as in several European countries [25], the WHO target for a functional dentition was not achieved.

## Conclusions

The majority of adults over 40 years presented a reduced dentition. Molars were more affected by decay and tooth loss than premolars and anterior teeth. Decayed, missing, filled teeth, and replaced teeth were associated with the background variables, however differently for different dental regions. Females appeared to have higher grade of utilization of dental care: higher chance for decayed and lower chance for missing teeth, but higher chance for filled teeth and tooth replacements. There were no distinct differences in decayed and missing teeth between urban and rural residents, but urban residents more often had filled teeth while rural residents more often presented tooth replacements. The WHO target for a functional dentition was not achieved: above the age of 70 years, approximately two-thirds of the subjects presented not less than 20 natural teeth. When counting teeth on the basis of natural plus replaced teeth, nine out of 10 subjects met this target.

## Competing interests

The authors declare that they have no competing interests.

## Authors' contributions

QZ carried out the data collection and drafted the manuscript. DJW was actively involved in designing the study, data interpretation and in manuscript writing. EMB carried out statistical analyses and participated in data interpretation. NHJC was actively involved in designing the study, data interpretation and in manuscript writing. All authors read and approved the final manuscript.

## Pre-publication history

The pre-publication history for this paper can be accessed here:

http://www.biomedcentral.com/1471-2458/11/420/prepub
